# *Caenimonas aquaedulcis* sp. nov., Isolated from Freshwater of Daechung Reservoir during *Microcystis* Bloom

**DOI:** 10.4014/jmb.2201.01023

**Published:** 2022-03-25

**Authors:** Ve Van Le, So-Ra Ko, Sang-Ah Lee, Mingyeong Kang, Hee-Mock Oh, Chi-Yong Ahn

**Affiliations:** 1 Cell factory Research Centre, Korea Research Institute of Bioscience and Biotechnology, Daejeon 34141, Republic of Korea; 2 Department of Environmental Biotechnology, KRIBB School of Biotechnology, University of Science and Technology, Daejeon 34113, Republic of Korea; 3 Environmental Safety Groups, Korea Institute of Science and Technology (KIST) Europe, Saarbrücken 66123, Germany

**Keywords:** Caenimonas aquaedulcis, Microcystis aeruginosa, freshwater, polyphasic characterization

## Abstract

A Gram-stain-negative, white-coloured, and rod-shaped bacterium, strain DR4-4^T^, was isolated from Daechung Reservoir, Republic of Korea, during *Microcystis* bloom. Strain DR4-4^T^ was most closely related to *Caenimonas terrae* SGM1-15^T^ and *Caenimonas koreensis* EMB320^T^ with 98.1% 16S rRNA gene sequence similarities. The average nucleotide identity (ANI) and digital DNA-DNA hybridization (dDDH) values between strain DR4-4^T^ and closely related type strains were below 79.46% and 22.30%, respectively. The genomic DNA G+C content was 67.5%. The major cellular fatty acids (≥10% of the total) were identified as C_16:0_, cyclo C_17:0_, summed feature 3 (C_16:1_*ω7c* and/or C_16:1_*ω6c*), and summed feature 8 (C_18:1_*ω7c* and/or C_18:1_*ω6c*). Strain DR4-4^T^ possessed phosphatidylethanolamine, diphosphatidylglycerol, and phosphatidylglycerol as the main polar lipids and Q-8 as the respiratory quinone. The polyamine profile was composed of putrescine, cadaverine, and spermidine. The results of polyphasic characterization indicated that the isolated strain DR4-4^T^ represents a novel species within the genus *Caenimonas*, for which the name *Caenimonas aquaedulcis* sp. nov. is proposed. The type strain is DR4-4^T^ (=KCTC 82470^T^ =JCM 34453^T^).

## Introduction

The genus *Caenimonas*, a member of the family *Comamonadaceae*, was first proposed by Ryu *et al*. [1] and amended by Kim *et al*. [2]. Since that formal description, the genus has encompassed three species with validly published names, including *Caenimonas koreensis*, *Caenimonas terrae*, and *Caenimonas soli*, and one non-validly published name species ‘*Caenimonas sedimenti*’ (https://lpsn.dsmz.de) [3]. Chemotaxonomically, members of this genus have ubiquinone-8 as major isoprenoid quinone and C_18:1_ *ω7c*, C_16:1_ *ω7c* and/or iso-C_15:0_ 2-OH, and C_16:0_ as main fatty acids [1]. The ecology of the genus *Caenimonas* has been reported from activated sludge [1], soil [2, 4], and sediment [5]. Additionally, they are highly distributed in mellow soil, suggesting their potential application for fertilizer production to enhance soil quality [6]. In this study, we used a polyphasic approach to ascertain the taxonomic position of strain DR4-4^T^ and proposed it as a novel species within the genus *Caenimonas*.

## Materials and Methods

### Strains and Culture Conditions

Strain DR4-4^T^ was isolated from a freshwater sample collected from the Janggye site, Daechung Reservoir, Korea (GPS location: 36° 22′ 33.7′′ N, 127° 38′ 20.6′′ E) in September 2019. The strain was successfully purified by re-streaking a single colony onto R2A plates. The stock cultures were preserved in R2A supplemented with 20%glycerol (v/v) at -80°C. For taxonomic analysis, *C. koreensis* EMB320^T^ (=KCTC 12616^T^), *C. terrae* SGM1-15^T^ (=KACC 13365^T^) *C. soli* S4^T^ (=KCTC 72742^T^), and ‘*Caenimonas sedimenti*’ HX9-20T (=KCTC 72473T) were selected as reference strains. All reference strains were procured from Korean Collection for Type Cultures (KCTC), except *C. terrae* KACC 13365^T^ obtained from Korean Agricultural Culture Collection (KACC). Since all strains could grow optimally on R2A, their chemotaxonomic and phenotypic features were characterized on R2A at 30°C after 3 days of incubation [7].

### Phylogenetic Analysis Based on 16S rRNA Gene Sequences

Genomic DNA was extracted from the pure cultures with FastDNA Spin DNA-extraction kit (MP Biomedicals). The 16S rRNA gene was amplified and sequenced using the universal primer set 27F (5’-AGAGTTTGATCATGGCTCAG-3’) and 1492R (5’-TACGGYTACCTTGTTACGACTT-3’) [8]. The nearly full-length 16S rRNA gene sequence of strain DR4-4^T^ (1,402 bp) was compared to those of valid species using the EzBioCloud server [9]. Phylogenetic trees derived from 16S rRNA gene sequence of strain DR4-4^T^ and related type strains were reconstructed by neighbor-joining (NJ) [10], maximum–likelihood (ML) [11] and minimum evolution (ME) [12] algorithms in MEGA X software [13]. The best substitution model for the ML tree was determined by the model test in MEGA X software with the lowest Bayesian information criterion scores. Accordingly, the ML tree was reconstructed using Kimura's two-parameter model [14] with a gamma distribution and invariant sites (K2+G+I). For the NJ and ME trees, the evolutionary distances were computed using the Kimura 2-parameter method [14]. The reliability of phylogenetic trees was estimated using a bootstrap procedure with 1000 replications.

### Genome Sequencing and Phylogenomic Analysis

Genome sequencing was performed on an Illumina MiSeq (Macrogen Inc.) and assembled *de novo* using SPAdes v3.12.0. Low-quality and adapter sequences were eliminated using Trimmomatic (v0.36) [15] to avoid biases in data analysis. The Benchmarking Universal Single-Copy Orthologous (BUSCO, v3.0) was used to measure assembly completeness [16]. The assembled genome was annotated by Prokka v.1.12 and RAST 2.0 (Rapid Annotation using Subsystem Technology) [17]. The putative secondary metabolite biosynthetic gene clusters in the genome of DR4-4^T^ were identified by the antiSMASH server [18]. The circular genomic map was visualized using PATRIC web service [19]. The phylogenomic tree of strain DR4-4^T^ and closely related taxa collected from GenBank database was constructed by the Type (Strain) Genome Server (TYGS) [20]. Average nucleotide identity (ANI) and digital DNA-DNA hybridization (dDDH) values were computed from whole-genome sequences using the ANI [21] and Genome-to-Genome Distance Calculator (GGDC 2.1) formula 2 [22], respectively. The two-way average amino acid identity (AAI) scores were obtained using the AAI calculator which was developed by Kostas lab (http://enve-omics.ce.gatech.edu/aai/).

### Phenotypic and Biochemical Analyses

Morphological characteristics of strain DR4-4^T^ were observed after growth on R2A for 3 days under a phase-contrast light microscope (Nikon Eclipse 80i) and a transmission electron microscope (CM20, Philips). Motility was tested using the hanging drop method. The Gram staining reaction was done with a Gram-stain kit (Becton Dickinson). Growth on different media was assessed using TSA, NA, and LBA (Difco) after 5 days of incubation at 25°C. The optimal pH for cellular growth was tested from pH 4.0 to pH 12.0 in intervals of 0.5 using the following buffer systems: phosphate-citrate (pH 4.0–6.5), Tris-HCl (pH 7.0–9.0), NaHCO_3_–NaOH (pH 9.5–11), and Na_2_HPO_4_–NaOH (pH 11.5–12). The effect of temperature on growth was assessed at 4, 10, 17, 20, 25, 30, 37, 40, and 60°C, respectively. The sensitivities of strain DR4-4^T^ to salt were tested in R2A supplemented with 0.5-11.0%sodium chloride in intervals of 0.5%. Growth was measured at an optical density of 600 nm. The presence of activity of oxidase and catalase in bacterial cells were detected using 1% (w/v) *N, N, N’, N*’-tetramethyl-1,4-phenylenediamine and 3% (v/v) H_2_O_2_, respectively. Starch, lipids, carboxymethyl cellulose, and skim milk hydrolysis were performed according to Smibert and Krieg [23]. Enzyme activities and production of acid from carbon source were determined using API ZYM kit and API 50 CH kit (bioMérieux), respectively, following the manufacturer’s instructions. Other biochemical tests were performed with API 20NE (bioMérieux). The sensitivity of strain DR4-4^T^ to antibiotics was performed using the Kirby-Bauer disc diffusion method [24] with antibiotic discs containing the following amounts (μg/disc): amikacin (30), ampicillin (10), amoxicillin (10), cefadroxil (30), cefoperazone (75), ceftazidime (30), ceftriaxone (30), chloramphenicol (30), ciprofloxacin (5), cloxacillin (1), erythromycin (15), gentamicin (10), nalidixic acid (10), netillin (30), nitrofurantoin (300), norfloxacin (10), penicillin(10), tobramycin (10), and vancomycin (30).

### Chemotaxonomic Analyses

For chemotaxonomic analysis, strain DR4-4^T^ and reference strains were grown at 30°C for 3 days. Cellular fatty acids were extracted according to Sasser [25], analyzed by gas chromatography, and identified using the TSBA 6 database of the Microbial Identification System. Polar lipid extraction from freeze-dried biomass was done according to Minnikin *et al*. [26]. The extracted polar lipids were separated on the thin layer chromatography (TLC) plates with chloroform/methanol/water (65:25:4, v/v/v) and chloroform/methanol/acetic acid/water (80:12:15:4, v/v/v/v) as mobile phases for the first and second chromatography dimension, respectively [27]. Total polar lipids were identified by staining with 5% molybdophosphoric acid in ethanol [28, 29]. Molybdenum blue, α-naphthol, and ninhydrin reagents were used to visualize phospholipids, glycolipids, and amino lipids, respectively. Respiratory quinones were extracted from agitating wet culture pellets using chloroform/methanol (2:1, v/v) for 3–4 h and analyzed by HPLC [30]. Polyamines were extracted and analyzed according to Busse and Auling [31].

## Results and Discussion

### 16S rRNA Phylogeny

**Fig. 1:**
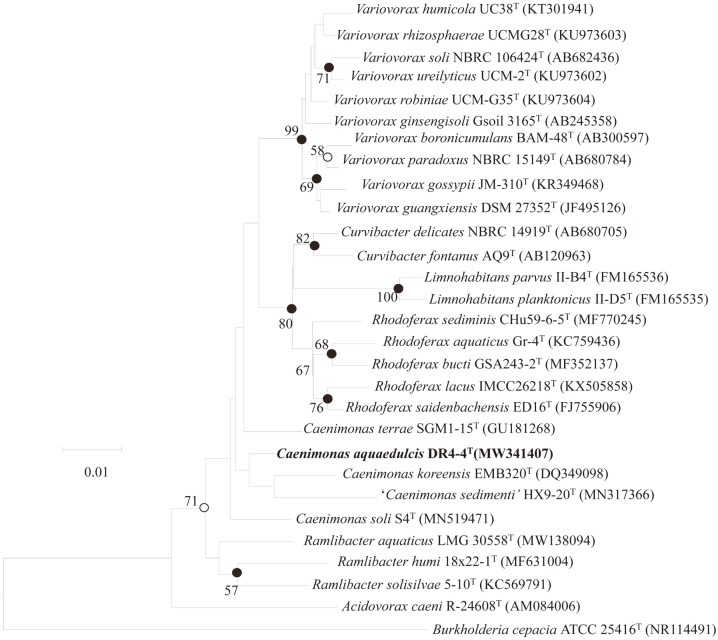
Neighbor-joining phylogenetic tree based on the 16S rRNA gene sequences showing the relationship of strain DR4-4^T^ to other members of the family *Comamonadaceae*. *Burkholderia cepacia* ATCC 25416^T^ (GenBank accession No. NR114491) was used as an outgroup. Bootstrap values (≥50%) based on 1000 replications were indicated at branch nodes. Filled circles at nodes indicate that the corresponding nodes were recovered in the trees reconstructed with three algorithms (NJ, ME, and ML methods) whereas the nodes with empty circles were recovered by two algorithms. Bar, 0.01 nucleotide substitutions per nucleotide position.

Based on 16S rRNA gene sequence similarity, strain DR4-4 T was most closely related to *C. terrae* SGM1-15^T^ (98.1%) and *C. koreensis* EMB320^T^ (98.1%). In phylogenetic trees, DR4-4^T^ formed a distinct branch within the genus *Caenimonas*, supporting the assignment of this strain to the genus *Caenimonas* (Figs. 1, S1 and S2).

### Genomic and Phylogenomic Analyses

The genome of strain DR4-4^T^ contained 3 contigs with a total length of 4,521,559 bp, an N50 length of 3,928,830 bp, and an L50 value of 1 (Fig. S3A). The completeness of the genome was 96.62%. The similarity level between 16S rRNA gene sequences retrieved from whole-genome data (1,523 bp) and that from Sanger sequencing (1,402 bp) was 100%, suggesting the authenticity of the genome assembly. The assembled genome sequence comprised 4,331 coding sequences, 43 tRNA genes, 1 tmRNA gene, and 3 rRNA genes. Most of the identified genes were involved in fundamental cellular processes such as metabolism, protein processing, energy, stress response, defense, and virulence (Fig. S3B). Microbes can produce secondary metabolites that play vital roles in interactions with their neighbors, such as competition, cooperation, and co-evolution [32, 33]. Notably, strain DR4-4^T^ possessed five putative secondary metabolite biosynthetic gene clusters responsible for the synthesis of terpene, arylpolyene, lassopeptide, nonribosomal peptide synthetase (NRPS), and NRPS-like, type I polyketide synthase (T1PKS) (Fig. S4). These compounds have been reported to be related to defense mechanisms [34-38]. Among the five predicted secondary metabolite regions, terpene and lassopeptide exhibited no similarity with any known gene clusters. This finding highlighted that strain DR4-4^T^ could produce such novel valuable natural compounds, which may be beneficial to competition.

**Fig. 2:**
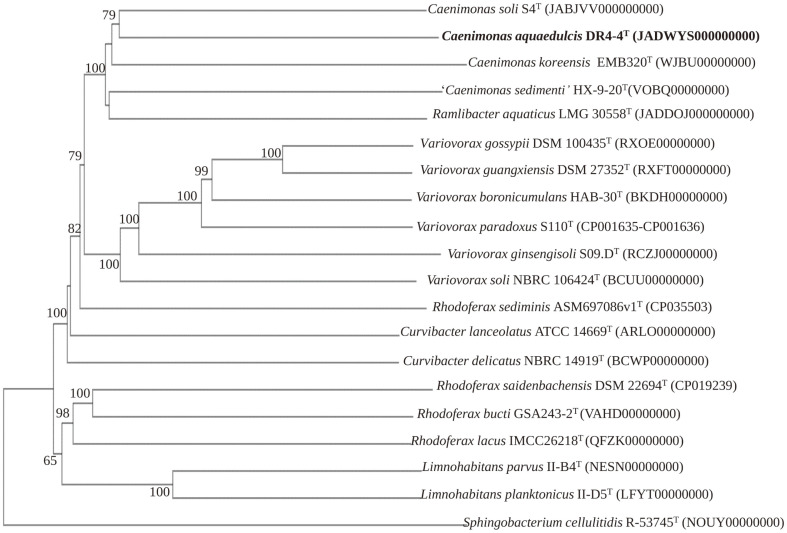
Phylogenomic tree constructed using Type (Strain) Genome Server (TYGS) showing the position of strain DR4-4^T^ among the species of the family *Comamonadaceae*. GenBank accession numbers are given in parentheses. The branch lengths are scaled in terms of GBDP distance formula d5. The numbers above branches are GBDP pseudo-bootstrap support values > 60% from 100 replications with an average branch support of 85.1%. The tree was rooted at the midpoint.

The draft genome sequence of strains DR4-4^T^ is publicly available on DDBJ/ENA/GenBank with accession number JADWYS000000000. The genomic G+C content of strain DR4-4^T^ was found to be 67.5%, falling within the range (62.7–68.7%) for *Caenimonas* species [2]. The phylogenomic tree indicated that strain DR4-4^T^ clustered closely with *C. soli* S4^T^ (Fig. 2). Strain DR4-4^T^ showed the highest AAI value with the type species of the genus *Caenimonas* (74.37%), followed by *Ramlibacter* (73.02%), *Variovorax* (66.56%), *Limnohabitans* (65.04%), *Curvibacter* (64.81%), and *Rhodoferax* (63.02%) (Table S1). These results suggested that DR4-4^T^ should be regarded as a member within the genus *Caenimonas* [39]. The ANI and dDDH values for strain DR4-4^T^ with its closely related type strains were below 79.46% and 22.3%, respectively (Table S2). Such values are much lower than the species boundaries of 95% for ANI and 70% for dDDH [40-43], suggesting the novel status of strain DR4-4^T^ within the genus *Caenimonas*.

### Phenotypic, Physiological, and Biochemical Characteristics

Strain DR4-4^T^ cells were Gram-stain-negative, non-motile, rod-shaped (0.3–0.5 μm × 2.5–4.5 μm) (Fig. S5), and positive for oxidase and catalase. The strain was unable to hydrolyze skim milk, Tween 20, Tween 80, carboxyl methylcellulose, and starch. Growth was observed on NA and R2A but not on TSA and LB media. Colonies formed on R2A were white, smooth, and convex with entire margins. The strain exhibited abundant growth at 30°C, pH 7.0, and in the absence of sodium chloride. DR4-4^T^ was susceptible to all tested antibiotics except cloxacillin. Strain DR4-4^T^ and reference strains shared many common phenotypic properties, such as non-motile, colony color, the activity of oxidase, and alkaline phosphatase. However, it could be differentiated from the closely related *Caenimonas* species by several biochemical characteristics such as hydrolysis of gelatin, producing enzyme lipase (C14), cysteine arylamidase, and trypsin (Table 1). Detailed comparison of phenotypic features of strain DR4-4^T^ with those of the closely related species is listed in Table 1 and Table S3.

### Chemotaxonomic Characteristics

In line with the description of the genus *Caenimonas*, the fatty acid profile of strain DR4-4^T^ was dominated (≥10% of the total fatty acids) by C_16:0_ (33.7%), cyclo C_17:0_ (21.1%), summed feature 3 (C_16:1_*ω7c* and/or C_16:1_*ω6c*)(22.0%) and summed feature 8 (C_18:1_*ω7c* and/or C_18:1_*ω6c*) (10.0%). Nevertheless, the ratios of some components such as C_14:0_, C_15:1_ *ω6c*, C_17:0_, and C_18:1_ *ω7c* 11-methyl are a key chemotaxonomic difference between DR4-4^T^ and references strains (Table 2). Strain DR4-4^T^ is distinct from *Variovorax* species with respect to the presence of iso-C_17:0_ 3-OH and C_18:1_ *ω9c* and the absence of C_12:0_ and C_18:0_ (Table S4). The fatty acid profile of *Curvibacter fontanus* AQ9^T^ differs from those of DR4-4^T^ by the presence of C_15:0_ as the predominant fatty acid (Table S4). The cellular fatty acid compositions of strain DR4-4^T^ and its close neighbor taxon are mentioned in Table 2 and Table S4. The polar lipid composition of strain DR4-4^T^ encompassed phosphatidylethanolamine, diphosphatidylglycerol, and phosphatidylglycerol (Fig. S6 and Table 1). Strain DR4-4^T^ is differentiated from *C. terrae* SGM1-15^T^, *C. koreensis* EMB320^T^, and *C. soli* S4^T^ by the absence of unidentified aminolipid. Additionally, phosphatidylcholine and three unidentified aminophospholipids were found in *C. sedimenti* HX-9-20^T^ but absent in strain DR4-4^T^. The respiratory quinone was Q-8, which is consistent with its affiliation as a member belonging to the genus *Caenimonas*. The polyamine profile of strain DR4-4 T was composed of putrescine 61.01%, cadaverine 33.86%, and spermidine 5.13% (Fig. S7), which is in accordance with the characteristic of members *Betaproteobacteria* [44]. Taken together, the data from phylogenetic, genomic, phenotypic and chemotaxonomic analyses supported that strain DR4-4^T^ should be considered as a novel species of the genus *Caenimonas*, for which the name *C. aquaedulcis* sp. nov. is proposed.

### Description of *Caenimonas aquaedulcis* sp. nov.

*C. aquaedulcis* (a.quae.dul’cis. L. fem. n. *aqua* water; L. masc. adj. *dulcis* sweet; N.L. gen. n. *aquaedulcis* of fresh water).

Cells are Gram-stain-negative, rod-shaped, approximately 2.5–4.5 μm in length and 0.3–0.5 μm in width. Oxidase and catalase-positive. Colonies grown on R2A after 3 days of incubation are white, smooth, and convex with entire margins. Growth is observed on R2A and NA at 10-37°C (optimally at 30°C) and at pH 7.0-7.5 (optimally at pH 7.0). It does not require NaCl for growth but can tolerate up to 0.5%. Negative for hydrolyzing skim milk, Tween 80, Tween 20, carboxymethyl cellulose, and starch. In the API ZYM system, alkaline phosphatase, esterase (C4), esterase lipase (C8), lipase (C14), leucine arylamidase, cystine arylamidase, trypsin, acid phosphatase, and naphthol-AS-BI-phosphohydrolase are positive but valine arylamidase, α–chymotrypsin, α-galactosidase, β-galactosidase, β-glucuronidase, α-glucosidase, β-glucosidase, N-acetyl-*β*-glucosaminidase, α-mannosidase, and α-fucosidase are negative. According to API 20NE test, the cells are positive for hydrolysis of esculin and gelatine but negative for reduction of nitrate to nitrite, reduction of nitrate to nitrogen, indole production, glucose acidification, arginine dihydrolase, urease, assimilation of D-glucose, L-arabinose, D-mannose, D-mannitol, N-acetyl-glucosamine, maltose, potassium gluconate, capric acid, adipic acid, malic acid, trisodium citrate, and phenylacetic acid. In the API 50CH system, a weakly positive reaction is observed for aesculin but not for the other substrates. The major cellular fatty acids are C_16:0_, cyclo C_17:0_, summed feature 3 (C_16:1_*ω7c* and/or C_16:1_*ω6c*), and summed feature 8 (C_18:1_*ω7c* and/or C_18:1_*ω6c*). The polar lipids are phosphatidylethanolamine, diphosphatidylglycerol, and phosphatidylglycerol. The respiratory quinone is ubiquinone Q-8. The polyamines are putrescine, cadaverine, and spermidine. The type strain, DR4-4^T^ (=KCTC 82470^T^ =JCM 34453^T^), was isolated from Daechung Reservoir. The DNA G+C content of the type strain is 67.5%. The GenBank/EMBL/DDBJ accession number of the 16S rRNA gene sequence and the whole genome sequence of type strain are OL587860 and JADWYS000000000, respectively.

## Supplemental Materials

Supplementary data for this paper are available on-line only at http://jmb.or.kr.

## Figures and Tables

**Table 1:** Differential characteristics of strain DR4-4^T^ and type strains of closely related species.

Characteristic	1	2	3	4	5
Catalase	+	+	-	+	+
Hydrolysis of Tween 20	-	+	-	-	-
**Growth range**					
pH	7.0-7.5	6.0-9.0^†^	5.0-8.0^#^	5.0-10.0^††^	6.0-8.0**
Temperature (°C)	10-37	10-35^†^	10-40^#^	10-37^††^	15-30**
**Enzymatic activity (API ZYM)**					
Lipase (C14)	+	-	-	-	-
Leucine arylamidase	+	+	-	+	+
Cystine arylamidase	+	-	-	-	-
Trypsin	+	-	-	-	-
**Other biochemical tests (API 20NE)**					
Reduction of nitrate to nitrite	-	-	+	-	+
Hydrolysis of:					
Esculin	-	-	-	+	+
Gelatin	+	-	-	-	-
**Polar lipids**	PE,PG, DPG	PE,PG, DPG,AL^†^	PE,PG, DPG, 3AL^#^	PE,2PL, AL,L^††^	PE,PG,PC, DPG,3APL**
**DNA G+C content**	67.5%	63.5%*	68.7mol%^#^	65.1%^††^	67.5%**

Strains: 1, DR4-4^T^; 2, *C. koreensis* KCTC 12616^T^ (^†^ data was obtained from Ryu *et al*., (2008) [1]; *data was obtained from wholegenome sequences data under accession number WJBU00000000); 3, *C. terrae* KACC 13365^T^ (^#^data was obtained from Kim *et al*., [2]); 4, *C. soli* KCTC 72742^T^(^††^data was obtained from Dahal *et al*., [4]); 5, ‘*Caenimonas sedimenti*’ KCTC 72473^T^(**data was obtained from Xu *et al*., [5]). PE, phosphatidylethanolamine; PG, phosphatidylglycerol; DPG, diphosphatidylglycerol; AL, unidentified aminolipid; APL, unidentified aminophospholipid; PL, unidentified phospholipids; PC, phosphatidylcholine; L, unidentified polar lipid. +, positive; -, negative; w, weakly positive reaction; NA, not available.

**Table 2:** Fatty acid contents (%) of strain DR4-4^T^ and type strains of closely related species.

Fatty Acids	1	2	3	4 ‡	5^§^
C_8:0_ 3-OH	-	-	-	4.4	-
C_10:0_	-	-	-	-	2.7
C_10:0_ 3-OH	4.6	3.7	2.8	3.4	2.6
iso-C_11:0_3-OH	-	-	-	1.3	-
C_14:0_	TR	1.1	TR	3.7	4.2
anteiso-C_14:0_	-	-	-	1.3	-
C_15:1_ *ω*6*c*	TR	3.3	-	-	-
C_16:0_	**33.7**	**22.0**	**35.6**	**22.9**	**24.3**
C_17:1_ *ω*6*c*	-	3.4	-	-	-
cyclo C_17:0_	**21.1**	-	**27.2**	5.4	5.6
C_17:0_	1.2	3.6	-	1.0	-
iso-C_17:0_ 3-OH	2.0	-	-	-	-
C_18:0_	-	-	-	1.0	-
C_18:1_ *ω*9*c*	1.0	TR	-	-	-
C_18:1_ *ω*7*c* 11-methyl	-	2.4	-	-	-
Summed Feature 3^*^	**22.0**	**44.5**	**19.8**	**29.1**	**46.6**
Summed Feature 7**	2.3	2.2	-	-	-
Summed Feature 8^†^	**10.0**	**10.3**	**12.0**	**14.5**	**10.6**
Summed Feature 9^††^	-	-	-	1.4	-

Strains: 1, DR4-4^T^; 2, *C. koreensis* KCTC 12616^T^; 3, *C. terrae* KACC 13365^T^; 4, *C. soli* KCTC 72742^T^ (‡Data was obtained from Dahal *et al*., [4]); 5, ‘*Caenimonas sedimenti*’ KCTC 72473T (^§^Data was obtained from Xu *et al*., [5]). Values are percentages of the total fatty acids. All data were obtained from this study, except where indicated otherwise. Major components (≥10.0%) are highlighted in bold. TR, Trace amount (<1.0%); −, not detected. * Summed Feature 3 comprises of C_16:1_*ω7c* and/or C_16:1_*ω6c*; ** Summed Feature 7 comprises C_19:1_ *ω6c*, C_19:0_ cyclo and/or an unknown fatty acid with an equivalent chain length of 18.846. † Summed Feature 8 comprises of C_18:1_*ω7c* and/or C_18:1_*ω6c*. Summed Feature 9^††^ comprises iso-C_17:1_*ω9c* and/or 10-methyl C_16:0_. Summed features are fatty acids that cannot be resolved reliably from another fatty acid using the chromatographic conditions chosen. The MIDI system groups these fatty acids together as one feature with a single percentage of the total.
