# Supplementary material for: Caenimonas aquaedulcis sp. nov., Isolated from Freshwater of Daechung Reservoir during Microcystis Bloom
Source: J Microbiol Biotechnol. 2022 Mar 25;32(5):575–81. doi: 10.4014/jmb.2201.01023 (PMC9628874; doi:10.4014/jmb.2201.01023)
Supplement: Supplementary file 1 [file jmb-32-5-575-supple.pdf]

J. Microbiol. Biotechnol.
https://doi.org/10.4014/jmb.2201.01023

Supplementary Materials

Caenimonas aquaedulcis sp. nov., Isolated from Freshwater of Daechung Reservoir during Microcystis Bloom

Ve Van Le[1,2], So-Ra Ko[1], Sang-Ah Lee[3], Mingyeong Kang[1,2], Hee-Mock Oh[1,2], and Chi-Yong Ahn[1,2*]

[1]Cell factory Research Centre, Korea Research Institute of Bioscience and Biotechnology, 125 Gwahak-ro, Yuseong-gu, Daejeon 34141, Republic of Korea

[2]Department of Environmental Biotechnology, KRIBB School of Biotechnology, University of Science and Technology, Daejeon 34113, Republic of Korea

[3] Environmental Safety Groups, Korea Institute of Science and Technology (KIST) Europe, Saarbrücken 66123, Germany

Table S1. AAI (%) values between strain DR4-4^T and type species of its closely related genera in the family *Comamonadaceae*

Species	Strain	Accession No.	AAI (%)
Caenimonas koreensis	EMB320	WJBU00000000	74.37
Variovorax paradoxus	S110	CP001635-CP001636	66.56
Rhodoferax fermentans	DSM 10138	NRRK00000000	63.02
Curvibacter gracilis	ATCC BAA-807	JADZ00000000	64.81
Limnohabitans curvus	MWH-C5	NESP00000000	65.04
Ramlibacter tataouinensis	TTB310	CP000245	73.02

Table S2. Genome comparison of strain DR4-4^{T} with reference genomes of related taxa

Species	Strain	Accession No.	Size (Mb)	G+C (%)	Gene	Protein	ANI (%)	dDDH (%)
Caenimonas aquaedulcis	DR4-4	JADWYS000000000	4.52	67.5	4380	4310	-	-
Caenimonas koreensis	EMB320	WJBU00000000	4.61	63.5	4252	4131	77.79	20.8
'*Caenimonas sedimenti*'	HX-9-20	VOBQ00000000	5.90	67.5	5741	5690	78.51	21.4
Caenimonas soli	S4	JABJVV000000000	5.64	65.1	5568	5517	79.46	22.3
Ramlibacter aquaticus	LMG 30558	JADDOJ000000000	4.12	70.2	4032	3988	78.39	21.5
Variovorax gossypii	DSM 100435	RXOE00000000	6.30	67.4	5855	5746	78.12	20.9
Variovorax guangxiensis	DSM 27352	RXFT00000000	7.18	67.4	6666	6559	78.14	20.9
Variovorax boronicumulans	HAB-30	BKDH00000000	6.70	68.4	6204	6108	77.90	20.8
Variovorax paradoxus	S110	CP001635-CP001636	6.76	67.5	6455	6431	77.84	20.8
Variovorax ginsengisoli	S09.D	RCZJ00000000	4.55	66.5	4279	4189	75.97	20.0
Variovorax soli	NBRC 106424	BCUU00000000	5.60	67.8	5379	5203	76.40	20.5
Rhodoferax sediminis	ASM697086v1	CP035503	4.39	64.3	4240	4090	76.17	20.1
Curvibacter lanceolatus	ATCC 14669	ARLO00000000	6.83	65.5	6330	6136	74.90	20.2
Curvibacter gracilis	ATCC BAA-807	JADZ00000000	6.75	66.0	6179	6035	75.18	20.2
Curvibacter delicatus	NBRC 14919	BCWP00000000	3.76	63.5	3656	3533	75.89	20.1
Limnohabitans planktonicus	II-D5	LFYT00000000	4.87	59.4	4138	3972	73.10	13.9
Limnohabitans parvus	II-B4	NESN00000000	2.97	59.2	2870	2785	73.03	19.0
Rhodoferax bucti	GSA243-2	VAHD00000000	3.67	61.2	3525	3453	73.14	18.5
Rhodoferax saidenbachensis	DSM 22694	CP019239	4.26	60.9	4053	3970	73.63	19.0
Rhodoferax lacus	IMCC26218	QFZK00000000	4.90	62.3	4562	4508	73.95	19.5
Ramlibacter solisilvae	5-10	CP010951	4.92	67.6	4581	4137	78.98	22.2

Table S3. Differential characteristics of strain DR4-4^T and type strains of related species

Characteristic	1	2†	3#	4††	5*	6**
**Growth range**						
pH	7.0-7.5	6.5-8.5	6.0-8.5	6.0-9.0	7	4.0-9.0
Temperature (°C)	10-37	4-35	4-35	10-37	20-30	4-35
**Enzymatic activity (API ZYM)**						
Alkaline phosphatase	+	+	+	+	+	-
Esterase (C4)	+	-	-	-	-	-
Esterase Lipase (C8)	+	+	-	+	-	-
Lipase (C14)	+	-	-	-	-	-
Cystine arylamidase	+	-	-	-	w	w
Trypsin	+	-	-	-	w	-
Acid phospatase	+	+	+	+	+	-
Naphthol-AS-BI-phosphohydrolase	+	-	-	-	+	+
**Other biochemical tests (API 20NE)**						
Reduction of nitrate to nitrite	-	-	-	-	+	-
Urease	-	-	-	-	+	-
Hydrolysis of:						
Gelatin	+	-	-	-	-	-
Assimilation of:						
D-Glucose	-	+	+	-	-	+
L-Arabinose	-	+	+	+	-	-
D-Mannitol	-	+	+	+	+	+
Potassium gluconate	-	+	-	+	+	-
Malic acid	-	+	-	+	-	+
**DNA G+C content**	67.5%	66.4 mol%	65.9 mol%	67.5 mol%	66.0-66.7 mol%	66.2 mol%

Strains: 1, DR4-4^T; 2, *Variovorax robiniae* UCM-G35^T († data was obtained from Nguyen *et al.*, [1]); 3, *Variovorax rhizosphaerae* UCM-G28^T (#data was obtained from Nguyen *et al.*, [1]); 4, *Variovorax ureilyticus* UCM-2 ^{T}(†† data was obtained from Nguyen *et al.*, [1]); 5, *Curvibacter fontanus* AQ9^T(*data was obtained from Ding *et al.*, [4]); 6, *Variovorax humicola* UC38^T (**data was obtained from Nguyen *et al.*, [3]).. +, positive; -, negative; w, weakly positive reaction; NA, not available.

Table S4. Fatty acid contents (%) of strain DR4-4^T and type strains of related species

Fatty Acids	1	2†	3#	4††	5*	6**
$C_{10:0}$ 3OH	4.6	3.0	4.5	1.7	5.3	2.1
$C_{12:0}$	-	3.1	4.0	1.7	-	3.0
$C_{14:0}$	TR	TR	2.9	-	4.1	-
$C_{15:0}$	-	-	-	-	**11.4**	-
$C_{15:1}$ *ω6c*	TR	-	-	-	3.1	-
$C_{16:0}$	**33.7**	**36.2**	**27.2**	**24.3**	**21.7**	**29.4**
$C_{17:1}$ *ω6c*	-	-	-	-	3.3	-
cyclo $C_{17:0}$	**21.1**	2.6	9.9	**18.2**	5.7	**16.2**
$C_{17:0}$	1.2	-	-	-	3.4	-
iso-$C_{17:0}$ 3-OH	2.0	-	-	-	-	-
$C_{18:0}$	-	1.5	1.0	1.0	-	1.3
$C_{18:1}$ *ω7c*	-	-	-	-	9.2	-
$C_{18:1}$ *ω9c*	1.0	-	-	-	-	-
Summed Feature 3*	**22.0**	**42.3**	**36.4**	**35.7**	**29.4**	**25.1**
Summed Feature 7**	2.3	-	-	-	-	-
Summed Feature 8†	**10.0**	**9.9**	**13.6**	**16.9**	-	**18.2**

Strains: 1, DR4-4^T; 2, *Variovorax robiniae* UCM-G35^T († data was obtained from Nguyen *et al.*, [1]); 3, *Variovorax rhizosphaerae* UCM-G28^T (#data was obtained from Nguyen *et al.*, [1]); 4, *Variovorax ureilyticus* UCM-2^T(†† data was obtained from Nguyen *et al.*, [1]); 5, *Curvibacter fontanus* AQ9^T(*data was obtained from Ding *et al.*, [4]); 6, *Variovorax humicola* UC38^T (**data was obtained from Nguyen *et al.*, [3]). Values are percentages of the total fatty acids. Major components (>10.0%) are highlighted in bold. TR, Trace amount (<1.0%); −, not detected. * Summed Feature 3 comprises of $C_{16:1}$*ω7c* and/or $C_{16:1}$*ω6c*; ** Summed Feature 7 comprises $C_{19:1}$ *ω6c*, $C_{19:0}$ cyclo and/or an unknown fatty acid with an equivalent chain length of 18.846. † Summed Feature 8 comprises of $C_{18:1}$*ω7c* and/or $C_{18:1}$*ω6c*.

Table S5. Negative reactions of strain DR4-4^T from the commercial test kits

API kits	DR4-4^T
**API ZYM**	valine arylamidase, α–chymotrypsin, α-galactosidase, β-galactosidase, β-glucuronidase, α-glucosidase, β-glucosidase, *N*-acetyl-*β*-glucosaminidase, α-mannosidase, and α-fucosidase
**API 20NE**	reduction of nitrate to nitrite, reduction of nitrates to nitrogen, indole production, glucose acidification, arginine dihydrolase, urease, assimilation of D-glucose, L-arabinose, D-mannose, D-mannitol, N-acetyl-glucosamine, maltose, potassium gluconate, capric acid, adipic acid, malic acid, trisodium citrate, and phenylacetic acid
**API CH50**	acid production from: glycerol, erythritol, D-arabinose, L-arabinose, ribose, D-xylose, L-xylose, adonitol, β-methyl-D-xylose, sorbose, rhamnose, dulcitol, inositol, mannitol, sorbitol, *N*-acethyl-glucosamine, arbutin, inulin, glycogen, xylitol, gentiobiose, D-turanose, D-lyxose, D-tagatose, D-fucose, L-fucose, D-arabitol, L-arabitol, gluconate, 2-keto-gluconate, 5-keto-gluconate, galactose, glucose, fructose, mannose, α-methyl-D-glucoside, α-methyl-D-glucoside, amygdalin, esculin, salicin, cellobiose, maltose, lactose, melibiose, sucrose, trehalose, melezitose, raffinose, and starch

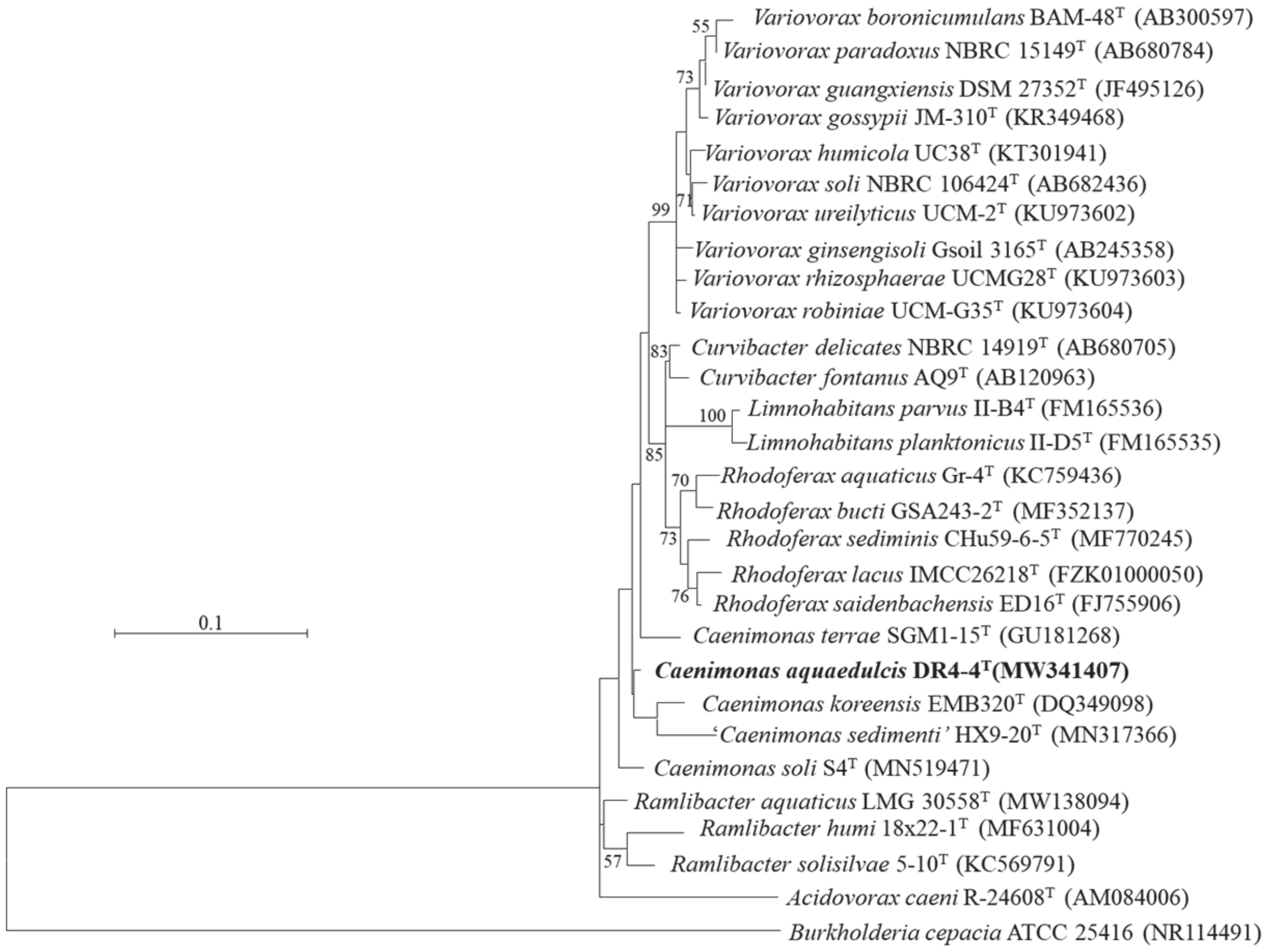

Fig. S1. Maximum-likelihood phylogenetic tree based on the 16S rRNA gene sequences showing the relationship of strain DR4-4^T to other members of the family *Comamonadaceae. Burkholderia cepacia* ATCC 25416 ^{T} (GenBank accession No. NR114491) was used as an outgroup. Bootstrap values (≥50%) based on 1000 replications were indicated at branch nodes. Bar, 0.02 nucleotide substitutions per nucleotide position.

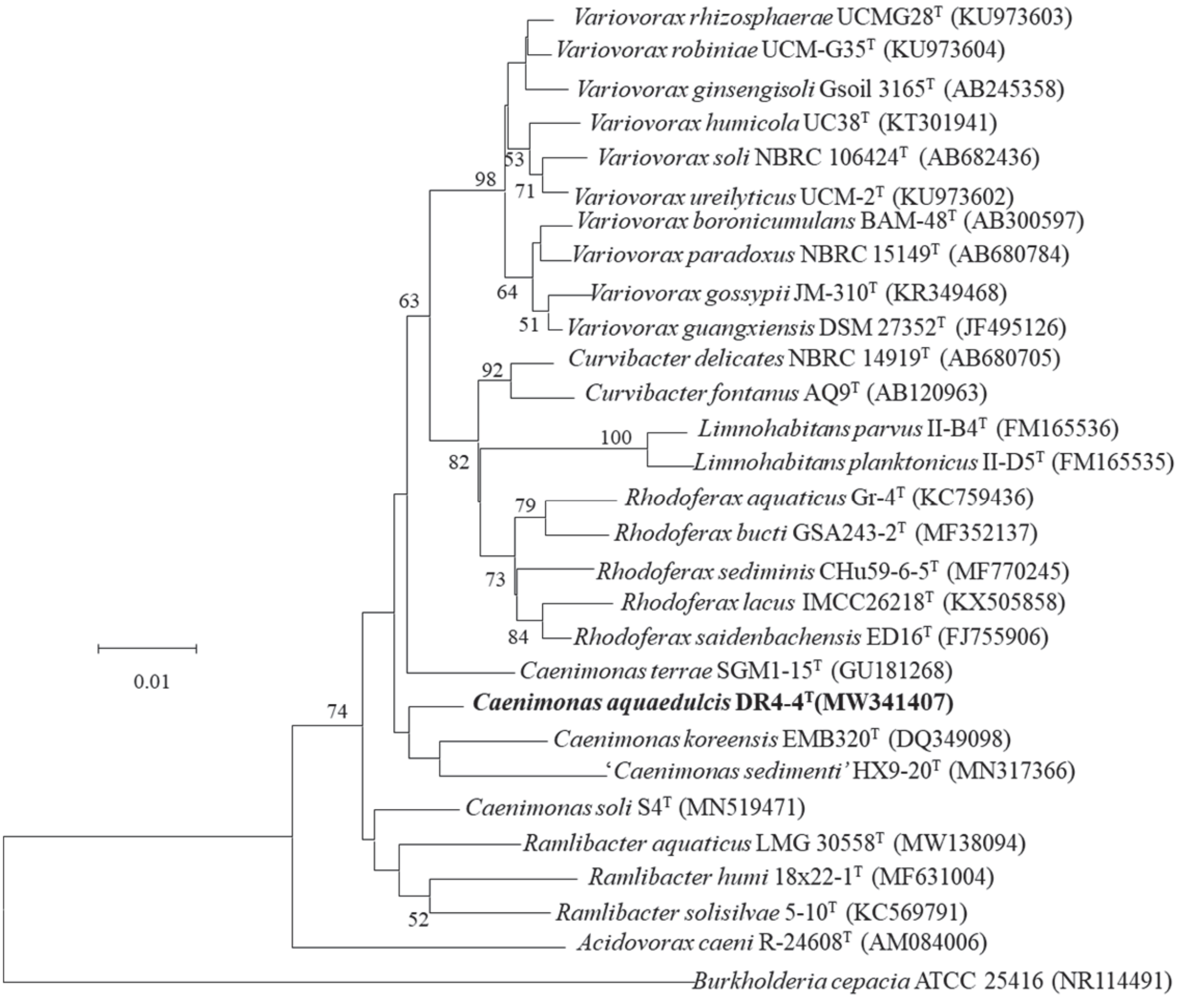

Fig. S2. Minimum evolution phylogenetic tree based on the 16S rRNA gene sequences showing the relationship of strain DR4-4^T to other members of the family *Comamonadaceae. Burkholderia cepacia* ATCC 25416 ^{T} (GenBank accession No. NR114491) was used as an outgroup. Bootstrap values (≥50%) based on 1000 replications were indicated at branch nodes. Bar, 0.02 nucleotide substitutions per nucleotide position.

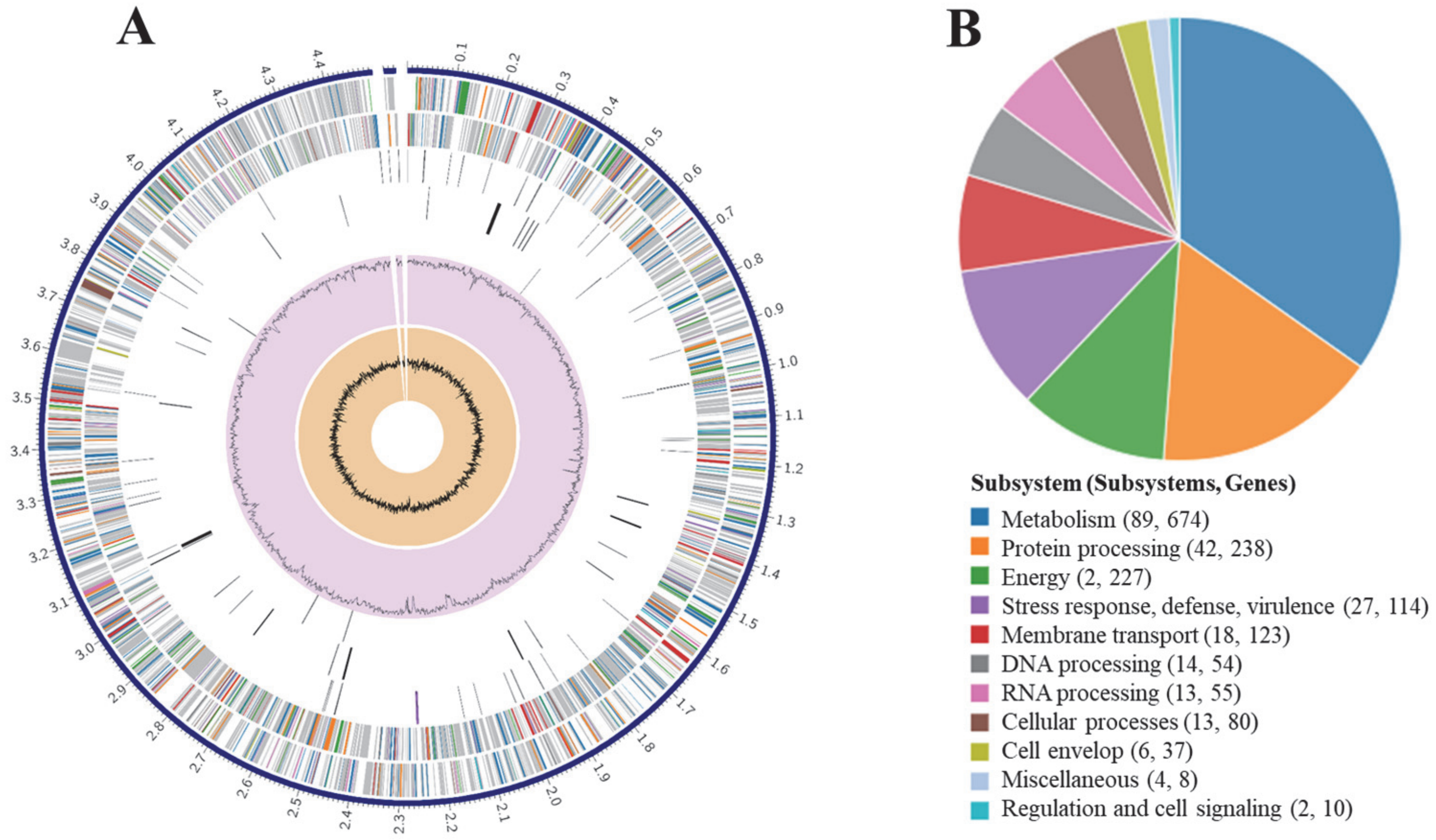

Fig. S3. Circular view (A) and features distribution (B) of the genome of strain DR4-4^{T}. (A) From the outside to the inside: contigs; coding sequence in forward strand; coding sequence in reverse strand; RNA genes; antimicrobial resistance genes; virulence factors;

GC content, and GC skew.

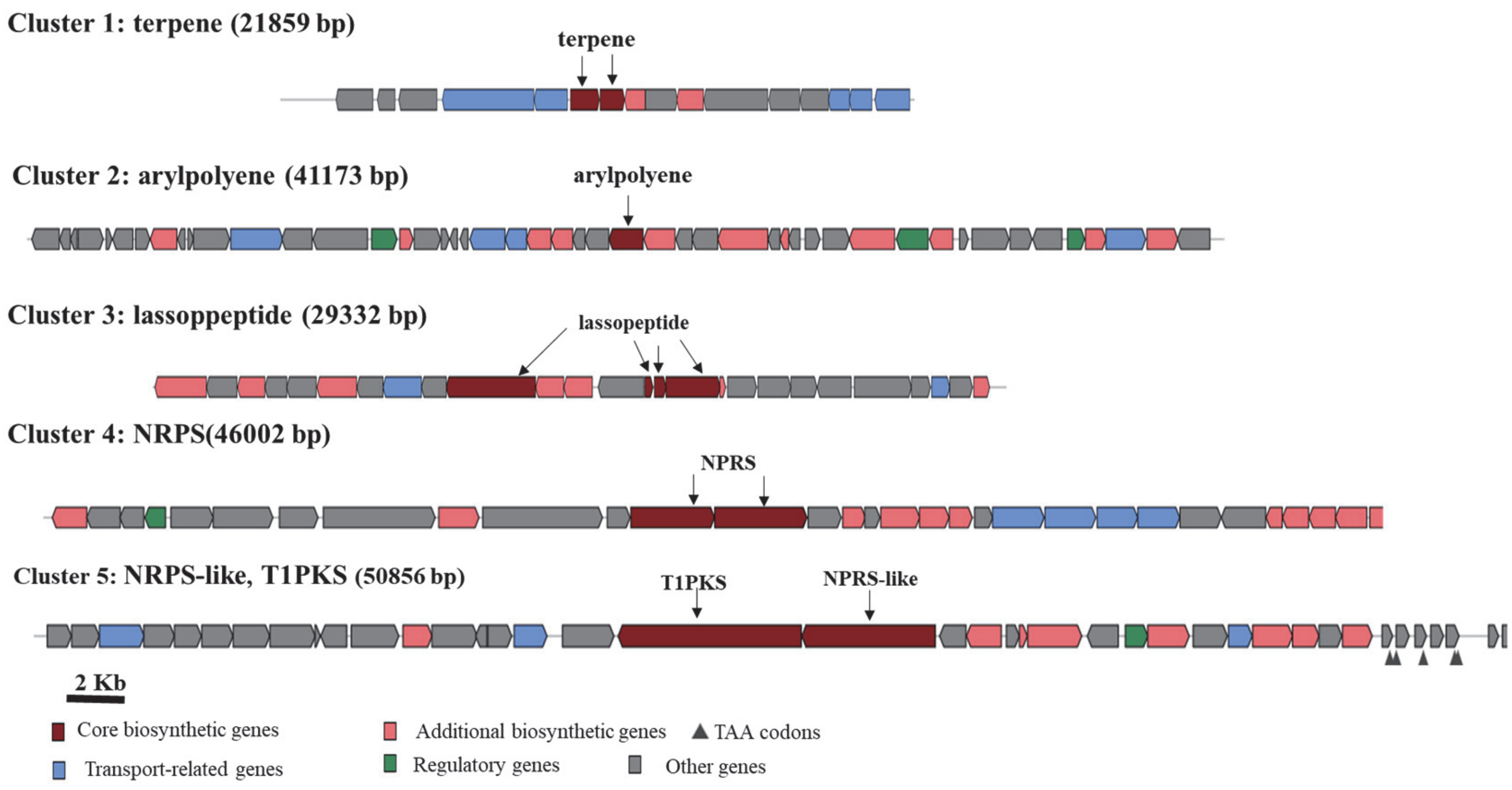

Fig. S4. Secondary metabolite biosynthetic gene clusters predicted in the genome of strain DR4-4^{T}

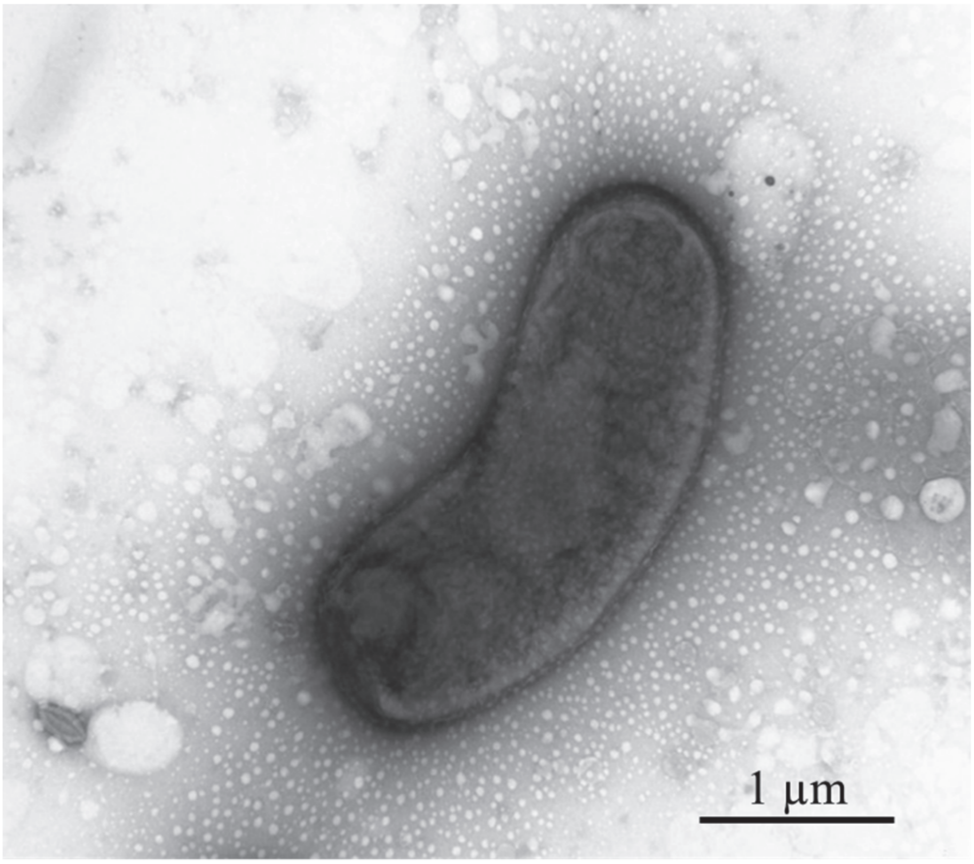

Fig. S5. Morphology of strain DR4-4^T. Transmission electron micrograph: bar, 1 μm. The cells were grown on R2A at 30°C for 3 days.

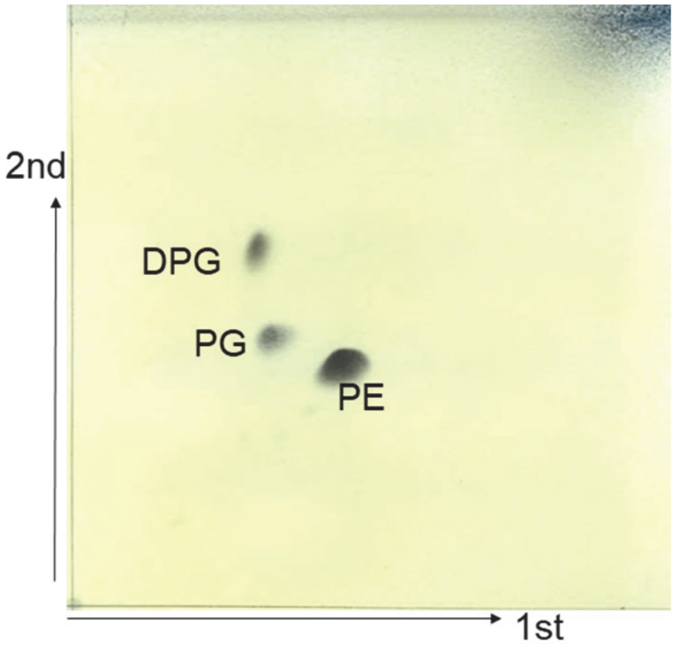

Fig. S6. Two-dimensional thin layer chromatography of polar lipids extracted from strain DR4-4^{T}. PE, phosphatidylethanolamine; PG; phosphatidylglycerol, DPG; diphosphatidylglycerol.

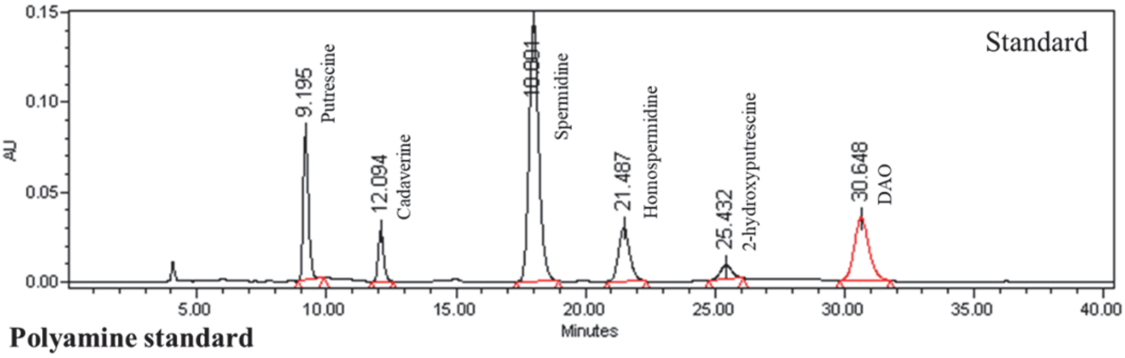

Polyamine standard

→ RT 9.195min. (Putrescine), RT 12.094min. (Cadaverine), RT 18.001min. (Spermidine),
RT 21.487 min.(Homospermidine), RT 25.432 min (2-hydroxyputrescine), RT 30.648min. (DAO)

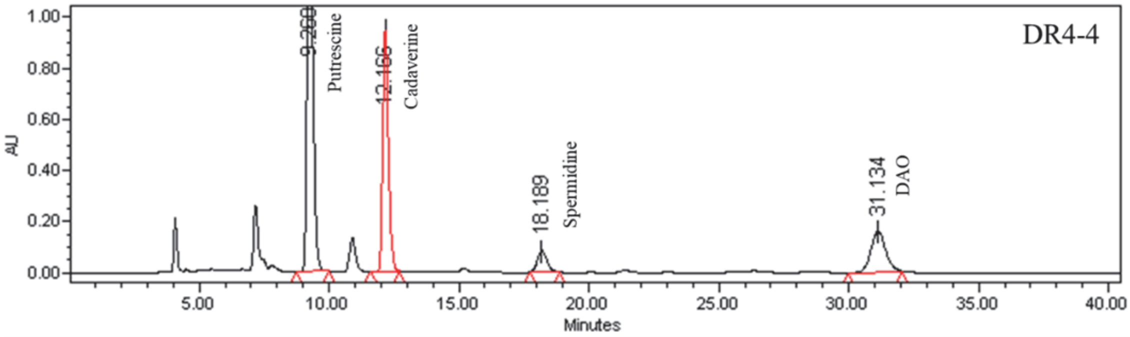

***Caenimonas* sp. DR4-4 polyamine**

→ RT 9.260min. [Putrescine(61.01%)], RT 12.166min. [Cadaverine(33.86%)], RT 18.189min. [Spermidine(5.13%)]

Fig. S7. Polyamine profile of strain DR4-4^T

References

1. Nguyen TM, Trinh NH, Kim J. 2018. Proposal of three novel species of soil bacteria, *Variovorax ureilyticus*, *Variovorax rhizosphaerae*, and *Variovorax robiniae*, in the family *Comamonadaceae*. *J Microbiol*. **56**: 485–492.
2. Zhou D, Tan X, Zhang W, Chen HY, Fan QM, He XL. 2019. *Rhodoferax bucti* sp. Nov., isolated from fresh water. *Int J Syst Evol Microbiol*. **69**: 3903–3909.
3. Nguyen TM, Kim J. 2016. Description of *Variovorax humicola* sp. nov., isolated from a forest topsoil. *Int J Syst Evol Microbiol*. **66**: 2520–2527.
4. Ding L, Yokota A. 2010. *Curvibacter fontana* sp. nov., a microaerobic bacteria isolated from well water. *J Gen Appl Microbiol*. **56**: 267–271.
